# Deep Learning for Chondrogenic Tumor Classification through Wavelet Transform of Raman Spectra

**DOI:** 10.3390/s22197492

**Published:** 2022-10-03

**Authors:** Pietro Manganelli Conforti, Mario D’Acunto, Paolo Russo

**Affiliations:** 1 DIAG Department, Sapienza University of Rome, Via Ariosto 25, 00185 Roma, Italy; 2 CNR-IBF, Istituto di Biofisica, Via Moruzzi 1, 56124 Pisa, Italy

**Keywords:** deep learning, raman spectroscopy, chondrogenic tumors, cancer tissues classification, CLARA

## Abstract

The grading of cancer tissues is still one of the main challenges for pathologists. The development of enhanced analysis strategies hence becomes crucial to accurately identify and further deal with each individual case. Raman spectroscopy (RS) is a promising tool for the classification of tumor tissues as it allows us to obtain the biochemical maps of the tissues under analysis and to observe their evolution in terms of biomolecules, proteins, lipid structures, DNA, vitamins, and so on. However, its potential could be further improved by providing a classification system which would be able to recognize the sample tumor category by taking as input the raw Raman spectroscopy signal; this could provide more reliable responses in shorter time scales and could reduce or eliminate false-positive or -negative diagnoses. Deep Learning techniques have become ubiquitous in recent years, with models able to perform classification with high accuracy in most diverse fields of research, e.g., natural language processing, computer vision, medical imaging. However, deep models often rely on huge labeled datasets to produce reasonable accuracy, otherwise occurring in overfitting issues when the training data is insufficient. In this paper, we propose a chondrogenic tumor CLAssification through wavelet transform of RAman spectra (CLARA), which is able to classify with high accuracy Raman spectra obtained from bone tissues. CLARA recognizes and grades the tumors in the evaluated dataset with 97% accuracy by exploiting a classification pipeline consisting of the division of the original task in two binary classification steps, where the first is performed on the original RS signals while the latter is accomplished through the use of a hybrid temporal-frequency 2D transform.

## 1. Introduction

The cancer disease with its diagnosis, analysis and investigation is still one of humanity’s greatest concerns. Improving the treatment specificity and developing tailored strategies on specific rare types of tumor is becoming more and more important thanks to the development of new performing technologies. It is noteworthy that among the different cancer typologies worldwide, less than 1% are primary bone tumors [1] and based on their frequency of appearance, they can be further divided into different subcategories: the osteosarcoma, which is one of the most frequently diagnosed sarcoma of bone (35.1%); followed by the chondrosarcoma (25.8%), the most frequent sarcoma of cartilaginous tissues; Ewing’s sarcoma (16.0%); and chordoma (8.4%) among others [2]. From a histological point of view, cartilagenous tissue tumors can be classified into four different categories: the enchondroma, which belongs to the family of benign tumors, and the three grades of cancerous chondrosarcoma tumors. The in vitro biopsy techniques typically allow for their differentiationbut require an experienced bone tumor pathologist for high accuracy responses. Additionally, they may have other operational limitations, such as a sensible diagnosis delay caused by the processing time of laboratory materials [1,3]. It is evident that the enchondroma-chondrosarcoma categorization represents an important step in correctly defining a clinical behavior and the associated therapies, which can significantly differ depending on the identified grade. Along with numerous other distinct biochemical criteria, the morphology of the tissue under investigation is the primary determinant of its classification.

Therefore, there is a sensible demand for the development of new, faster and more accurate classification technologies which would eventually combine recent advancements on different fields into single solutions, such as artificial intelligence (AI) and medical imaging processing techniques.

Potential beneficial advancements include increases in classification procedure accuracy, decreases in analysis costs, diagnostic times, and test invasiveness, as well as the creation of new hardware and software to assist health workers.

Raman spectroscopy (RS) is a promising, label free, non-destructive technique [4] able to determine the biochemical attributes of tumor tissue; the spectra it provides are great indicators for the analysis and monitoring of several diseases located in different body parts [5,6,7,8], thus making the application of RS an important challenge for automated medical analysis.

The development of new, more efficient AI-based approaches and a remarkable growth in the accessibility of data resources contributed to the advancement of computing capabilities over the past ten years. Large datasets actually allow us to train more sophisticated and resource-intensive models, such as the Convolutional Neural Networks (CNN) [9,10], the Recursive Neural Network (RNN) architectures [8] and the Transformer Network [11,12]. Moreover, the transfer of the learned knowledge (also known as *transfer learning*) leads to new possibilities of inter-disciplinary research in many different fields, e.g., medical images analysis and healthcare. Applied to medical tasks, these techniques produced results that were comparable to or even outperformed those of human experts [13].

In this work, we propose a *chondrogenic tumor CLAssification through wavelet transform of RAman spectra* (CLARA), a deep learning technique that can identify enchondroma tumor grades and the early stages of chondrosarcoma with a high degree of confidence starting from their Raman spectra. We trained and tested CLARA on the same dataset used in [4], which is composed of RS samples belonging to 10 patients in different chondrogenic tumor stages: the benign enchondroma(E) and three malignant grades, first grade (G1), second one (G2) and third one (G3), respectively. CLARA uniqueness lay in the special combination of a hybrid 1D-2D deep learning classification process applied on Raman spectra both raw and after wavelet transform. To the best of our knowledge, this approach has never been employed for tumor classification. Our method’s ability to operate with raw data without the need for labor-intensive processing or artifact removal processes while also ensuring a high level of classification accuracy is one of its key advantages. The performances obtained by CLARA are unbiased and much faster than a human investigation, leaving openings for future advancements in the field. Moreover, it addresses the never ending growing necessity for automatic analysis to support the medical diagnosis procedure of rare diseases.

The rest of the paper is organized as follows: the related works Section 2 addresses similar use cases of Raman spectroscopy followed by an in depth comparison on medical diagnosis applications performed with the help of machine learning and deep learning techniques. Section 3 introduces the novel elements and approaches of our work, while Section 4 describes the networks implementation details, the proposed data augmentation schema and the hyperparameters tuning. Finally, all the results will be discussed and examined in Section 5, with the final considerations reported in Section 6.

## 2. Related Works

The RS is a vibrational spectroscopy technique used to determine the characteristic vibrational states of observed molecules. The aim of this technique is to discover the chemical composition of different kinds of materials with an approach that is at the same time label-free and non-destructive. Recent advancements in many fields such as electronics, laser and optics, as well as in computer science and artificial intelligence, have made the RS technique a very promising approach for measuring the biochemical composition of materials, with many additional potential applications. For instance, one interesting RS field of application can be found in the food and drugs analysis, where RS can be of great use to optimize quality controls in industrial processing up to a molecular level. This can be achieved by directly analyzing the product chemical composition for quality testing [14] or to detect anomalies, as happened with the adulterated Suichang native honey [15]. Concerning the industrial area, RS can be of great assistance for the analysis of product packaging, with the example of Yun Peng et al. [16] who perform a real time and in situ analysis of plastic packaging. Another domain of use of this technique is in the materials science, where it can be used to comprehend the behavior of complex energetic microstructures [17], as in graphene electrical conductivity in [18] or in an in situ investigation on carbon materials in batteries [19]. Moreover, a very important field to consider is environmental science, wherein the RS technique can be used to monitor pollution as performed by T. Somekawa et al. in [20] for a quantitative analysis of subsea oil leaks from pipelines. In this task, an RS-based method was able to detect but also prevent oil spill accidents. Another example can be found in [21], where the problem of microplastics inside the global ocean system is addressed with an introductory approach for the field to RS for a qualitative and quantitative analysis of marine microplastics and nanoplastics.

The medical diagnosis is another essential area of use for the RS technique. By extracting information regarding the molecular makeup of the examined tissue, the RS can greatly assist the physician’s disease diagnosis. The RS’s ability to deliver real-time, qualitative, in situ valuations of living tissue is one of its most intriguing features [22], other than being incorporated in many medical devices, e.g., endoscopes, cannulas and needles. However, it can also be applied on ex vivo tissues [23].

Research works on RS under investigation are wide ranging. For instance, this approach can be used to monitor blood components, as described in [24,25]. Here, the blood analytes concentrations can be of critical importance (e.g., glucose is a critical indicator of diabetes, while blood serum concentrations of urea and creatinine may indicate renal dysfunction). RS can also be of great help for discovering and monitoring diseases of various part of the body, for instance in the brain (e.g., with Alzheimer’s disease [26]) or in the bones (e.g., concerning atherosclerosis and the related atherosclerotic plaques [27]), thanks to the described properties.

With the use of biomedical technology, significant scientific work is currently being conducted on the detection and eradication of neoplasias. Cancers are one of the most investigated and deadliest diseases and one of the most influential for exploiting healthcare-related RS implementations.

Machine learning techniques are frequently used to correctly classify tumors because of their ability to find an underneath scheme of the chemical components of the cancerous tissue and understand its biological characteristics. In the research carried out by Shangyuan Feng et al. [28], a surface-enhanced Raman spectroscopy method was involved in the blood plasma analysis for a non-invasive nasopharyngeal cancer detection test. Two groups of patients (about 70 in total), composed of healthy or with confirmed nasopharyngeal carcinomas people, were examined in order to analyze cancerous patterns inside the data, obtaining great discriminative results. To conduct the study and achieve this goal, the two strategies used were the principal component analysis (PCA) [29] in combination with the Linear discriminant analysis (LDA) [30]. Similar machine learning approaches can be found in [7], where the PCA output is used together with the discriminant function analysis method or with a support vector machine (SVM) algorithm in order to distinguish and classify RS samples of breast cancers. Diagnosis and classification of gastric cancer has been performed by Seng Khoon Teh et al. [31] where RS samples of 53 patients are used to identify chemical changes of tissue related to gastric cancer transformation. The statistical analysis is performed by the use of a classification and regression tree technique.

According to the chemical differences and the complexities behind the grades of the observed tumor, more recent deep learning approaches are able to improve the expressive ability of traditional machine learning techniques, as the latter may not properly encode the nonlinear effects [32]. Many research studies in the questioned field obtain remarkable results through these kinds of techniques, exploiting ever increasing computational power and data resources.

Concerning bone tumors, which are the subject of our research, several technologies have been used in recent years to conduct an AI-oriented diagnosis. Most traditional sources of information are based on radiological data, e.g., X-ray, SPECT, CT, MRI, or on pathological images, e.g., a histopathologic analysis of the tumor tissue images [33]. These can be used singularly or in a combined manner in order to obtain a more robust system. In the work [34], an MRI-based DL analysis is performed in order to predict the malignancy of bone tumor samples, exploiting two pre-trained ResNet50 architectures. Further patient demographics information is then combined together with the network outputs and given to a fully connected layer to obtain the final prediction. Another radiological-based DL analysis is conducted in [35], where samples of standard anteroposterior hip radiographs are exploited in order to classify autonomously the considered bone tumors. Several preprocessing steps are employed to stabilize and strengthen out the predictions of the evaluated architectures (ResNet, GoogleNet and EfficientNet). A SPECT-based automated diagnosis of bone metastases is examined in [36], where SPECT bone images containing the thoracic region of different patients are analyzed. The sample images are cropped and augmented after a denoising procedure in order to properly highlight and extract the patient’s thoracic area information from the original samples. Afterwards, multiple traditional DL classifiers are analyzed in the proposed classification task, including VGG, ResNet and DenseNet, obtaining promising results.

Concerning the pathological image analysis, in [37] ML and DL-based investigations are performed, which regard the bone tumor necrosis rate after chemotherapy treatments in order to monitor the patients response to the therapy. In the work, a set of glass slide samples of the tumor are first digitized into whole slide images, then a selection of them is magnified and subdivided into smaller sections in order to generate a dataset. Among the several AI-methods compared, the best result is achieved through a custom convolutional neural network trained from scratch.

In addition to the possible technologies described for the bone tumor analysis, deep learning approaches based on spectroscopy techniques (such as the RS) are considered by [33] as a novel and promising field, oriented in the direction of a new, encouraging path for the autonomous identification of the aforementioned tumors.

Focusing on the analysis of tumors through RS samples from a DL point of view, we can divide the different approaches mostly into two groups, one referring to custom architectures trained *from scratch* and the other regarding proven popular architectures (e.g., the use of Alexnet, GoogLeNet or Resnet in [9]) used in a transfer learning setting.

Concerning the first subset, common approaches mainly use: Artificial Neural Networks (ANN), one (or two)-dimensional Convolutional Neural Networks (CNN) or Recursive Neural Network (RNN). In the first approach, the 1D signal is usually given to an ANN after the data preprocessing, as to augment data and filter out defective spectra with a wide range of different techniques. An example concerning skin cancer has been carried out in [38], where an estimated background spectra is subtracted from the original signal spectrum in order to obtain a more reliable suppressed sample. Then, the input is reduced by the use of a PCA, and an ANN is used for the classification.

CNN is a standard approach when dealing with two-dimensional inputs but also a common choice for one-dimensional signal. Many works through these kinds of architectures extract the space-related features for a variety of RS tumor specters [5,6,32,39]. Other approaches exploit the RNN temporal knowledge to aim for better results, as in the case of [8], where all the types of discussed architectures (ANN, CNN and RNN, in particular with an LSTM architecture) are compared.

More in general, adapting architectures that already exist and are performing well in different fields of application can lead to great results. This can be achieved by exploiting the already existing and proved architecture features (e.g., the residual mechanism for ResNet, or the attention mechanism for the vision transformer) to then modeling from scratch the network structure on the specific observed data. Moreover, the knowledge obtained while training the network on very large quantities of data can be independently useful. For instance, ImageNet [40] contains 1000 different classes; therefore, a large variety of features have to be extracted in order to classify them correctly. A network pre-trained on this dataset, as performed by Young-Gon Kim et al. in [10], exhibits better results than the random initialized one in classifying tumor images. Lastly, as we can expect, when a domain-specific dataset is available the transfer learning procedure is more efficient, as in the latter case of [10], where the pretraining on Camelyon16 dataset [41] shows more improvement in the results than the ImageNet-based ones.

## 3. The CLARA Method

CLARA is a cancer classification technique operating on the Raman spectra, making it possible to distinguish benign tumors from first grade and second-third grade cancers. More in detail, with CLARA we propose the following pipeline:

- The RS retrieved from a tissue sample is normalized and pre-processed, in order to regularize and generalize the informative contents of the spectra.
- The elaborated signals go through a deep, one-dimensional model which acts as a binary classifier between the E, G1 versus the G2, G3 aggregated categories.
- The samples recognized as malignant grades are transformed through a hybrid time-frequency transformation, producing an RGB two-dimensional (2D) image.
- The image is given as input to a pre-trained deep model for 2D image classification between the benign tumor class and the first grade cancer category.

**Figure 1:**
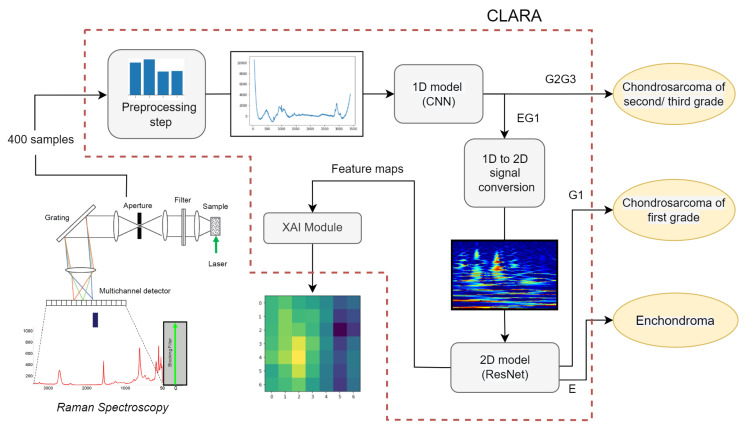
CLARA pipeline, from the extraction of Raman Spectroscopy from sample tissue to the analysis of 1D and 2D signals, which gives as output the sample tumor category.

Figure 1 shows the proposed classification process. We would like to remark that the first classification can be of great utility to guide surgical management to address the right diagnosis, while the CS grade can be an excellent predictor of cancer clinical behaviour. This is because the metastasis risk for grade G1 is significantly lower than grade G2 and G3, thus the following treatment can differ significantly. Moreover, there are distinct biological traces in the chemical composition of the two subset which can be exploited by a machine learning procedure, leading to very high accuracy without requiring human intervention.

However, because G3 cancers frequently still exhibit G2 area, the grade assignment method is dependent on the specific tissue sample, making the distinction between G2 and G3 grades less significant. For this reason, we focus on a three categories (E, G1 and G2G3) classification, leaving the G2, G3 classification to a future work. The rest of the section is organized as follows: in Section 3.1 we explain in detail the procedure to retrieve the RS from tissue samples. In Section 3.2 we show the deep model implemented in the first classification phase, performed directly on the processed Raman spectroscopy. In Section 3.3 we explain the Raman 1D signal conversion to a 2D RGB image through the use of wavelet transform. Finally, in Section 3.4, we show the details of the proposed deep model which is able to classify between E and G1 categories by taking as input the 2D image obtained in the previous step.

### 3.1. Raman Spectroscopy

The RS analysis was carried out with a Thermo Fisher Scientific DXR2xi Raman microscope. A total of 10 patients were analyzed, who were being treated at Institution, Azienda Ospedaliera Universitaria Pisana, Pisa under Ethical Committee agreement. Details of the case analyzed can be found in [4].

**Figure 2:**
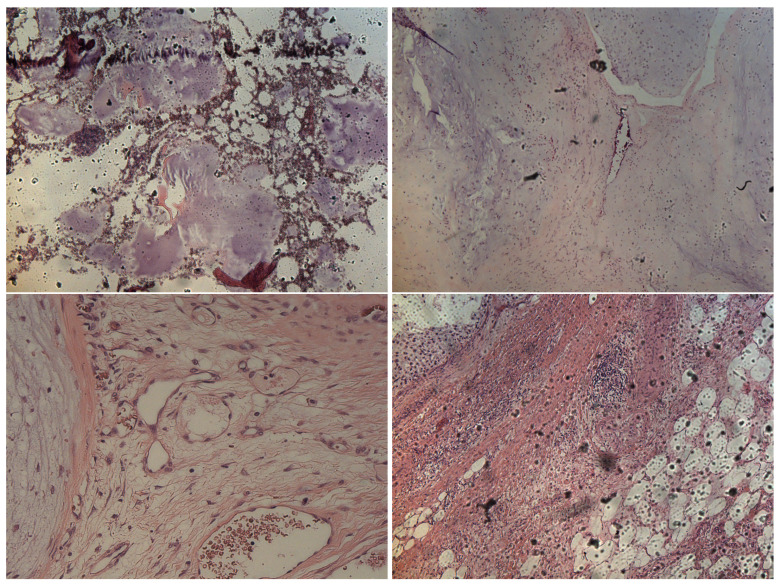
Representative histological images of samples belonging to E (upper left image), G1 (upper right image), G2 (lower left image) and G3 (lower right image) categories, respectively.

Formalin-fixed paraffin-embedded tumor tissue sections (e.g., in Figure 2) were collected on glass slides and subsequently submitted to RS analysis after the de-paraffination step. The RS measurements exploited a configuration based on the following experimental parameters: laser wavelength 532 nm; power laser of 5–10 mW; 400–3400 cm${}^{-1}$ full range grating; 10×, 50× and 100× objectives; 25 µm pinhole; 5(FWHM) cm${}^{-1}$ spectral resolution. Integration time for recording a Raman spectrum was 1 sec and 10 scans for any spectrum. As a first step, the tissue morphology overview was carried out to identify the regions of interest with the collection of a number of mosaic images at low (10×) and intermediate (50×) magnification. Thus, the acquisition of Raman spectra was carried out with a 100× objective. Optimization of signal-to-noise ratio and minimization of sample fluorescence were obtained through preliminary measurements in order to set the best experimental parameters. Multiple measurements were performed in different regions within the various samples, in order to assess intra-sample variability. In turn, no pretreatment of the samples was necessary before Raman measurements. Minimal preprocessing, including background removal and baseline application, was performed by using the tools of the DXR2xi GUI. Peaks were identified with specific tool support by Omicron 9.0 software.

Raman hyperspectral chemical maps ranging from 50 × 50 µm${}^{2}$ (step size 1µm) to approximately 200 × 200 µm${}^{2}$ (step size 4 µm), recording several hundreds of spectra per map were collected. Raman maps provide the fundamental advantage of being able to localize Raman spectra to specific locations, providing local information about chemical composition. Step sizes were chosen to have a collection time for each map less than 7 h for all the maps.

The RS to such chondrogenic tissues evidenced progressive degradation of collagen from EC to CS G3 (strong Raman bands at 728 cm${}^{-1}$, 830 cm${}^{-1}$ and 1206 cm${}^{-1}$) and analogously for chondroitin sulfate (strong Raman band at 1380 cm${}^{-1}$). This degradation is not surprising because collagen and chondroitin sulfate are the primary components of cartilaginous tissues. Another evidenced result of applying RS are the increasing of DNA/RNA signals as the malignant degree is increased due to cellular proliferation. In turn, CS G1 highlighted calcified areas as evidenced by the rich presence of hydroxyapatite (Raman band 960 cm${}^{-1}$), easily identified from the topography in the correspondent optical images. All such results can be found in [4], where the Raman range under investigation was limited to 400–1800 cm${}^{-1}$. On the contrary, here, we consider the full recorded raw signals with range 200–3400 cm${}^{-1}$, including artifacts such as the tail of Rayleigh scattering, to show the robustness of CLARA, which is able to ignore noise, artifacts and uninformative signal portions while extracting visual features which correspond to the most important signal parts.

### 3.2. 1D Classification

In the CLARA pipeline, we perform a preliminary classification which aims at separating with high accuracy the whole dataset into two subsets: one containing the benign stage of the tumor together with the first grade of its malignant stage (EG1) and the other one holding the second and third cancer grades (G2G3). With this procedure, we can effectively split the data in relation to the gravity of the tumor samples while simplifying the task, making the sub-problem of E, G1 classification addressable with a dedicated deep network.

In order to achieve this first classification, we apply a specifically tailored one-dimensional Convolutional Neural Network (1-D CNN), comparing it with several machine learning models usually employed in the literature for signal classification (e.g., ANN, SVM, LDA). The normalized one-dimensional spectrum acquired from Raman spectroscopies serves as the input data for each of these algorithms after being enhanced by various data augmentation techniques often employed for 1D signal analysis. They include a left/right shift function and different additive noise types that are absolute or proportional to the peaks intensities. Both are used to address the data scarcity and possible slight biological differences in both intensities and location of the RS peaks of the examined sample tissue. Furthermore, the data is normalized with zero mean and unit variance to get better algorithms performance and stability. As will be seen in the Results section, the best architecture for the first classification step is a CNN with a single convolutional layer with kernel size of (1, 4, 6), followed by a maxpool operator with kernel size of (3,3) and by three fully connected layers, with size of 1024, 256 and 128, respectively. A batch norm layer operation is added after each fully connected layer to improve the training stability and convergence. The one-dimensional value obtained after a final sigmoid activation function is then used to predict the input sample category, between ‘EG1’ or ‘G2G3’.

### 3.3. Signal 1D-2D Conversion through Wavelet Transform

In many signal processing applications, a multidimensional analysis on both time and frequency components can be fundamental to highlight specific spectral features. Moreover, to exploit the powerful visual features learned by pre-trained deep neural networks, 3 channels (RGB) 2-D input data are required. For these reasons, we opted for a two-dimensional time-frequency signal representation. Among the several available decomposition methods, we considered the *Short Time Fourier Transform (STFT)*, a traditional signal processing technique used in many application domains. In the STFT, the Fourier transform decomposes the signal into sine waves of multiple frequencies and is applied repeatedly to small time windows in order to obtain the frequency components for each time segment. At this point, the results are combined into a 2D scalogram in both time and frequency domain, as performed in [42]. A limitation of the STFT is the time (or frequency) resolution, which concerns the time window applied; however, it can also be continuously changed during the procedure to still obtain a valid multi-resolution analysis. While the STFT uses a single time-window size, the *Continuous Wavelet Transform* (CWT) can use short windows at higher frequencies and long windows at lower frequencies [43]. Thus, it is a valid alternative if a *non-stationary* signal has to be analyzed to produce the corresponding time-frequency representation (TFR), as explained in [44]. For instance, we can find CWT application in a variety of biomedical applications, for common signal processing analysis [45] but also concerning AI-oriented classifications (e.g., ECG [9] or EMG [46]). The mathematical formulation (Equation (1)) is defined given a positive scale parameter *a*, a translation value *b*, a mother wavelet function Ψ(t) and an input signal s(t) [47]:

(1) $$W_{s}\left(a,b;f\left(t\right)\right),\Psi \left(t\right))=\frac{1}{\sqrt{a}}\int_{-\infty }^{\infty }s\left(t\right)\Psi^{*}\left(\frac{t-b}{a}\right)dt$$

where ψ* is the complex conjugate of the mother wavelet ψ and $W_{s}$ the time-frequency (or time-scale) representation of the signal. *Wavelets* are finite energy and rapidly decaying oscillatory signals which exists for a finite duration. The main purpose of the mother wavelet function is to generate the daughter wavelets according to the scale and translation values. When analyzing distinct frequency components, the scaled length of the wavelets can be of great utility. For example, longer daughter wavelets at low frequencies can enhance frequency investigations at the expense of time localization, and shorter wavelets at high frequencies can do the opposite [48]. Thus, modifying these two parameters leads to a complete time-frequency analysis.

According to the formula above (1), various mother wavelets can produce significantly different TFRs depending on the input data used, and can thus highlight different patterns within them. Moreover, complex wavelets can be exploited in the CWT analysis because they separate phase and amplitude components associated with the signal. In our case, the complex General Morse (mother) Wavelet (*gmw*) obtained the best accuracy results for our goal.

**Figure 3:**
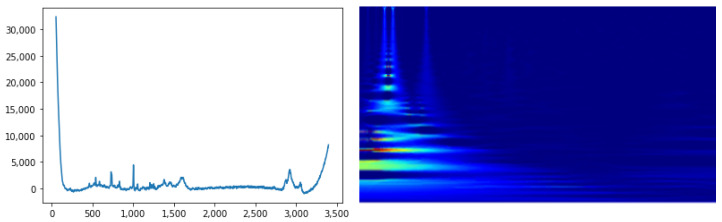
Representative 1D RS sample on the left and the corresponding synchrosqueezed CWT on the right.

A last but important step is the *synchrosqueezing* wavelet transform procedure. Starting from the $W_{s}\left(a,b;f\left(t\right)\right)$, the instantaneous frequencies are extracted using a phase transform. Then, the $W_{s}$ regions are modified (“squeezed”) where the same phase transform is constant. The final aim is to sharpen the input TFR, reassigning the values to different points in the TFR plane in relation to their local behavior, as described in [49]. An interesting aspect is that both CWT and the synchrosqueezed version have the invertibility property, therefore the original signal can be reconstructed. An example of Raman signal and the corresponding synchrosqueezed CWT can be seen in Figure 3.

### 3.4. 2D Classification

The second classification task aims to distinguish with high accuracy the enchondroma samples, with label ’E’, from first grade chondrosarcomas, with label ‘G1’.

Due to the lack of data and the substantial more challenging task, in CLARA we propose a pre-trained deep CNN which takes as input the 2D images representing the synchrosqueezed CWT transform of our 1D samples. The 2D images in RGB format are provided by a simple colormap function (JET) applied on the grayscale output of the CWT. The applied *transfer learning* technique provided a boost in the performances thanks to the powerful feature representations learnt on the 1000-category classification task on ImageNet dataset.

The ResNet18 [50] deep model has been chosen as CLARA 2D classifier; it is a 18 layers deep convolutional neural network based on a residual learning architecture, which greatly improves the network convergence and the resilience to overfitting. Moreover, it has been successfully exploited in many applications in the medical field [9,10].

### 3.5. Baseline Methods

In order to assess CLARA performance, we implemented some machine learning techniques as baseline, applying them to the full three-category classification task and using them as alternative algorithms in the CLARA pipeline.

**SVM**: the Support Vector Machine is a supervised algorithm originally conceived for binary classifications of data points belonging to high-dimensional input spaces; it finds the optimal hyperplane that divides the data into two categories. However, by using a one-vs-one method among all classes, it may be applied to multi-class problems. For this reason, we employed it both as an alternate technique in the CLARA pipeline and as a single, three-category classifier.

**LDA**: the Linear Discriminant Analysis algorithm is a generalization of Fisher’s linear discriminant [51] which aims at finding a linear combination of features that separates two or more categories. Although it assumes that data come from a multivariate Gaussian distribution and that the task categories share the same covariance parameters, it often works with good accuracy even when the assumptions are violated. LDA makes predictions by estimating the likelihood that a new set of inputs belongs to each class after finding the hyperplanes that maximize the separation between the classes. It can be effectively used both as a dimensionality reduction algorithm or as a classifier; we employed it as a classification algorithm as it has been exploited to differentiate the tissues types also by the original RS dataset authors [4].

**ANN**: the Artificial Neural Network, also called Multi Layer Perceptron, is a classical, shallow neural network model which is able to find non linear patterns in the data by interleaving neuron layers, which perform a weighted sums of the input, with the activation functions adding non-linearities to the system. It is a supervised learning technique which we be trained through back-propagation algorithm as in CLARA.

**CNN**: the Convolutional Neural Network is an improvement of the classical ANN where each neuron is substituted by a kernel convolved with the input data. The convolutional operation significantly reduces the amount of learnable parameters compared to ANN, which makes CNN less prone to overfitting issues. Furthermore, CNN is able to learn robust and general features by applying the learned filters to different parts of the input signal in order to recognize underlying data patterns. As a CNN baseline method, we propose the same CNN model currently presented in CLARA, in order to demonstrate the effectiveness of 1D + 2D CNN classification as opposed to two 1D CNNs.

**ResNet, EfficientNet, SENet**: As extensions of the generic CNN, many specific architectures are commonly employed in order to improve the system capabilities with well performing network from other fields. In our case, we considered the following CNN architectures:

The *residual neural network* [50] (also known as ResNet), which is a popular neural network often employed as a backbone for many computer vision tasks. It has been introduced to solve the vanishing gradient problem which arises during CNN trainings, and to further increase the overall accuracy. The architecture is composed of a series of layers grouped as residual blocks. The critical feature inside each residual block is the skip connection (or identity connection), which allows a better gradient flow during backpropagation by adding the block input directly to the output. There are many variants of this network, in relation to the number of layer implemented; for CLARA, we have chosen the ResNet-18 variant with its 18 layers due to the small number of trainable parameters and thus the increased robustness to overfitting.

The *EfficientNet* architecture [52] is a CNN-based model which introduces the concept of compound coefficient. When fine-tuning the network size, the compound scaling method equally adjusts the dimensions using a predetermined fixed set of scaling factors. By balancing the width, depth, and resolution while taking into account a constant ratio, the overall accuracy and efficiency of the network is sensibly enhanced.

An evolution of the residual model is proposed in the *Squeeze-and-Excitation Network* (also known as SENet), which leverage a novel squeeze and excitation block. Firstly, a squeeze procedure is conducted to aggregate the knowledge outside the local receptive fields of convolutional filters, using a global average pooling operator. Next, an excitation operation is performed employing a self-attention mechanism made by two fully connected layers, used to weight the squeezed information. In the end, the channel-wise input features are weighted by the outputs of this procedure, to increase the network sensitivity to global information.

It has to be noticed that the SVM and LDA methods are applied after a dimensionality reduction step performed with the PCA algorithm. PCA works by calculating the principal components of high dimensional data and by re-projecting the data to a lower-dimensional data space. However, we empirically found that it helps the SVM technique but hinders the performances of LDA, thus we report LDA performances both with and without PCA pre-processing step. Lastly, a comparison of the 2D architectures was conducted in order to evaluate the variations in the system outputs in relation to a lighter model, such as the selected EfficientNet, a medium-sized model, such as ResNet, and the computationally expensive SENet-154.

## 4. Implementation Details

The reported implementation details mainly concern three important aspects: the dataset samples manipulation/generalization, the full system hyperparameters fine tuning and the aggregated accuracy formulation resulting from the combination of the two classifiers.

### 4.1. Dataset

As previously stated, the aim of this work is to go through the chondrogenic tumor classification problem introducing a new applicable solution for further studies. In this context, we propose the RS as the main feature extractor of raw information from the patient tissues. Indeed, the dataset used to assess the validity of CLARA is composed of the 400 RS signals taken from the tissues of 10 patients, each belonging to one of the three possible categories (E, G1, G2G3). We used a one-patient per class out split technique to compose the training and test splits which have been used both for CLARA and for the baseline methods. This splitting approach ensures that the single patient samples do not show in the training and test subsets at the same time, which would make the task way more prone to overfitting. However, the number of samples belonging to each category is not balanced: the E-G1-G2G3 categories contains 69-76-101 samples in the training set, and 31-24-99 E-G1-G2G3 samples in the test set, respectively. For this reason, a *weighted random sampler* has been applied during the training so that each sample comes from each class with equal probability, thus guaranteeing a better balancing in the dataset. Different types of data augmentation techniques have been used in order to properly generalize the data, in both the 1D and 2D scenarios. The best result has been achieved through an additive noise sampled from a normal distribution of zero mean and standard deviation proportional to the peak value. Due to potential inaccuracies in the wavelength calibrations, small shifts in the spectra are also exploited to increase the network robustness. For each sample, a positive or negative shift is considered, with a shift value sampled from an uniform distribution over the [*−s, s*] interval. A value of *s* equal to 5 is used in the experiments, while high values of *s* (i.e., *s* ≥ 10) led to a deterioration in the performances of the algorithm.

In the case of 2D classification task, we also applied a random cropping function which allows for slight adjustments in the 2D signal, producing a maximum shift of the 2% of the axis lengths. Then, the resize operation bring back the cropped signal to the standard ratio of (224,224) required for the chosen pre-trained network [50]. Moreover, the 2D samples are normalized according to their mean and standard deviation, stabilizing the training through the z-score normalization.

Finally, a Gaussian noise with zero mean and 0.1 standard deviation, together with an image sharpness adjuster, is employed to increase the network generalization ability similarly to the 1D case.

### 4.2. Hyperparameters

The CLARA 1D CNN has been trained with a batch size of 32 and a learning rate of 0.0001 using the Adam optimizer with betas = (0.9, 0.999) and eps = $10^{-8}$. To stabilize the training, a weight decay value of 0.01 has been applied. The 2D classification task has been performed through the use of a pre-trained on ImageNet ResNet18 network, where the last fully connected layer has been replaced by one with the number of neurons equal to the number of task categories (two or three). A dropout technique with *p* = 0.2 has been implemented in order to further reduce overfitting. Other parameters considered are a batch size equal to 64 and a learning rate of $5\times 10^{-7}$ with the same optimizer function and parameters of the 1D case.

For both 1D and 2D networks, the binary cross entropy and the categorical cross entropy functions have been used for two-classes and three-classes tasks, respectively.

Regarding the baseline techniques employed, the LDA algorithm uses a singular value decomposition solver with a threshold value equal to 0.0001 and without shrinkage. The SVM considered a radial basis function as kernel, with a gamma coefficient inversely proportional to the input size times its variance. The regularization parameter C is kept to 1. Both methods were also tested considering a PCA reduction technique with a number of principal components equal to 30. Finally, concerning the synchrosqueezed continuous wavelet transform (*ssq_cwt*), we considered a generalized Morse wavelet (“gmw”) as the mother wavelet function Ψ, jointly with an increased number of the voices (nv = 64) and with a ‘log’ scale, to improve the visual variance of the output images.

### 4.3. Accuracy

The CLARA classification system is based on the application of two classifiers which act on three categories with an asymmetric approach. Indeed, the samples recognized as G2G3 (which we treat as a single category) by the first 1D neural network do not undergo any additional classification, while the samples recognized as EG1 are subjected to a second classification step performed by the 2D deep neural network. For this reason, the final aggregated accuracy over the three categories can be calculated starting from the per-class accuracy of the single networks.

We define $Acc_{E}^{f}$, $Acc_{G1}^{f}$ and $Acc_{G2G3}^{f}$ as the final per-class accuracy of, respectively, E, G1 and G2G3 categories over the full dataset. Furthermore, we define $Acc_{EG1}^{1}$ and $Acc_{G2G3}^{1}$ as the per-class accuracies obtained by the 1D neural network acting as a binary classifier over the two categories EG1 and G2G3. Finally, $Acc_{E}^{2}$ and $Acc_{G1}^{2}$ are the per-class accuracies of E and G1 categories calculated by using the 2D deep neural network on the samples previously classified as E or G1. Given those definitions, the final mean accuracy $Acc^{f}$ over the three categories E, G1 and G2G3 on the full dataset is expressed by Equation (2):

(2) $$Acc^{f}=\frac{n_{E}Acc_{E}^{f}+n_{G1}Acc_{G1}^{f}+n_{G2G3}Acc_{G2G3}^{f}}{n_{E}+n_{G1}+n_{G2G3}}$$

where $n_{E}$, $n_{G1}$ and $n_{G2G3}$ are, respectively, the number of dataset samples belonging to the E, G1 and G2G3 categories.

The final per-class accuracy of $Acc_{G2G3}^{f}$ is simply equal to the per-class accuracy $Acc_{G2G3}^{1}$ obtained by the first classifier:

(3) $$Acc_{G2G3}^{f}=Acc_{G2G3}^{1}$$

while in the case of $Acc_{E}^{f}$ and $Acc_{G1}^{f}$ the results come by performing a multiplication with the respective first classifier accuracy:

(4) $$Acc_{E}^{f}=Acc_{E}^{2}*Acc_{EG1}^{1}$$

(5) $$Acc_{G1}^{f}=Acc_{G1}^{2}*Acc_{EG1}^{1}$$

## 5. Results

This section reports the results obtained by CLARA both in its full pipeline and in its separated components. Furthermore, CLARA is compared with the baseline methods presented in Section 3.5, applied in two variants: in the first one (*three-classes*, denoted as *3c*) the classifiers are directly employed in a single, three categories task; in the second (*pipeline*, denoted as *p*) the algorithms perform the same classification schema of CLARA. This gives the possibility to understand the contribution of the proposed pipeline as well as the single networks.

As can be seen from the values reported in Table 1, CLARA outperforms all the tested baseline methods. In particular, the mean accuracy obtained by our method is almost 15 and 10 percentage points greater than the LDA^1^ method and the plain 1D CNN, respectively, which are the two most performing baseline methods. These algorithms struggle to produce a satisfying accuracy both when used in the straightforward three-classes classification and when employed in the CLARA pipeline, although in this latter case the neural networks techniques (ANN and CNN) are able to get a modest increase in performance. This demonstrates the effectiveness of our method, whose strength lies in a peculiar pipeline applied to powerful but lightweight networks. As relates the per-class performances, we can notice that the E and G2G3 categories show a high accuracy in all the methods, with only LDA struggling in the identification of E samples; however, the G1 category is strongly mis-classified by all methods but CLARA, with a difference of more than 50 points of accuracy with respect to the best baseline technique (LDA). As previously reported, the classification of G1 category is crucial as the medical treatment of this cancer stage is vastly different both with respect to tumor E than with respect to tumor G2 and G3, and in this regard CLARA is the only technique producing a satisfiable accuracy.

To better understand the contribution of the two binary classification tasks to the overall procedure, Table 2 and Table 3 show the CLARA 1D CNN and the CLARA 2D CNN performances, respectively, comparing them with the accuracies obtained by the other methods applied on the same pipeline. The results clearly demonstrate that the G2G3 is the easiest category to be classified, with all the accuracy values equal to 100% but one. Again, most of the challenges regards the recognition of G1 samples, with poor results produced by all the baseline techniques. Instead, the ResNet18 deep CNN is able to reach up to 83.3% of accuracy, which explains the high final mean value. The added complexity of SENet model [53], with its increased number of trainable parameters along the squeeze and excitation blocks, does not correspond to an increase in accuracy, which is slightly inferior with respect to the ResNet18 value. Moreover, the EfficientNet model [54] is producing a sensible lower accuracy, suggesting that its optimized and lightweight architecture does not give any advantage in the CLARA pipeline. Please notice that all the methods are subjected to overfitting issues due to the dataset samples coming from a limited amount of patients. Further studies will be performed on additional data to understand the impact of data on the enchondroma-chondrosarcoma classification task; still, we can hypothesize inferior generalization capabilities of shallow methods with respect to CLARA CNN models, which particularly shine when big amount of training data is available, while we could expect possible better results with the SENet and the EfficientNet models.

### Explainable AI

The explainable AI field (XAI) is becoming progressively more important due to the increased demand for artificial intelligence applications. While most of the deep learning approaches are characterized by “black-box” results, XAI techniques try to explain the reasons and the underlying mechanisms which lead to the model outputs. In particular, in this subsection, we show the preliminary results of the *Class Activation Maps* (CAM) technique [55] applied to CLARA 2D CNN features. CAM produces an image heatmap by weighting the importance of each feature map used by the last classification layer; then, the application of the heatmap as an overlay to the input image highlights the image areas most responsible of the classification. In the case of CLARA we applied CAM to identify the areas in the 2D scalograms that are mostly responsible for the E-G1 classification.

**Figure 4:**
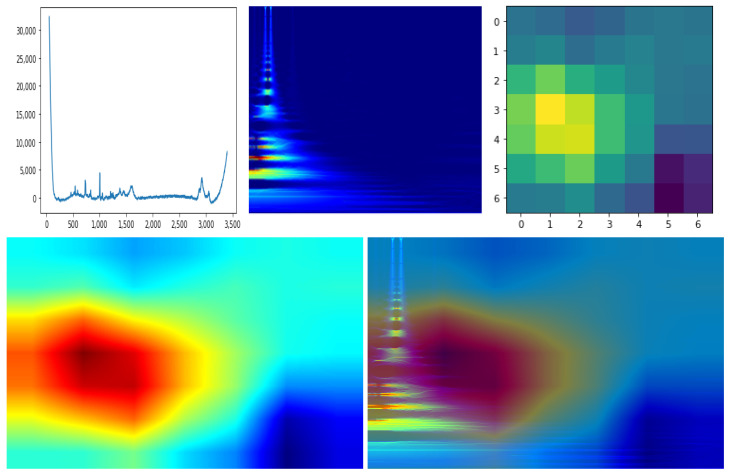
Application of CAM procedure on a single sample. The up-left image refers to the 1D RS plot, while the up-center image is the representation of that signal in the transformed 2D space. The up-right image is the 7 × 7 CAM output, which is smoothed and mapped to a different color palette through the JET color function for easier visualization (low-left image). Finally, the processed CAM image can be merged with the 2D signal image (low-right image) to qualitatively check the signal parts areas most responsible of the sample classification.

An example of CAM application on CLARA is reported in Figure 4. We extracted from a single sample the 7 × 7 CAM which can be blended with the 2D input image after a colormap transformation which enhance the CAM visualization. As explained in Section 3.3, the 1D signal has an approximate horizontal correspondence to the 2D spectrogram due to the mathematical operation (see Equation (1)). Thus, we can notice the correspondent salient interval on the 1D signal that highlights its most important section for the network. The result shows that the RS frequencies most responsible for the classification are the ones up to ∼1800, which corresponds to the RS frequency cutoff suggested in [4]. However, we would like to remark that this result still cannot be generalized due to the low amount of dataset samples. Further experiments on a bigger set of data are required in order to formally establish the most significant RS frequency patterns for all tumor categories, as well as to put strict frequencies cutoffs and to detect signal artifacts which can be automatically discarded as uninformative and/or misleading.

## 6. Conclusions

This paper presented CLARA, a enchondroma-chondrosarcoma classification technique exploiting Raman spectroscopy signals as input. CLARA demonstrated high accuracy (97%) when applied on the dataset under investigation, outperforming the most common baseline methods. CLARA has been trained on a 400 RS samples dataset, which was demonstrated to be big enough for deep learning training thanks to a two-step classification pipeline which divided the main task in two binary classification procedures, including the four malignant degrees of enchondroma-chondrosarcoma considered in our study. The one-dimensional classification makes it possible to distinguish between the tumor’s early stages and more advanced ones with great accuracy. The subsequent two-dimensional analysis improves the classification process and diagnosis by exploiting a module that can confidently distinguish between the tumor’s malignant and non-cancerous stages. However, the system is still limited by the small quantity of available samples; for this reason, further studies will focus on the development of a bigger datasets with an increased number of patients to be recruited. Moreover, additional efforts will be necessary to confront different types of architectures and possible 2D transformations, being a crucial part of the system. Finally, the XAI preliminary study will be expanded with a focused, in-depth investigation, in which several approaches will be examined along with a full-invertible transformation to enable the accurate localization of the frequencies that are most important for the right classification procedure.

## 7. Ethics Statements

The study was approved by the local Ethical Committee *Comitato Etico Regionale per la Sperimentazione Clinica della Regione Toscana sezione AREA VASTA NORD OVEST* (protocol number 14249). Ten patients affected by primary chondrogenic tumors of the skeleton were enrolled in this study. Informed consent was collected from all patients. All the experiments were carried out in accordance with Good Clinical Practice (GCP) and with the ethical principles of the Declaration of Helsinki. All patients were diagnosed and treated at Azienda Ospedaliera Universitaria Pisana, Pisa, in 2018.

## Figures and Tables

**Table 1:** CLARA final accuracy results, compared with the most common baseline models. The first three rows show the per-class accuracy, while the last row reports the total mean classification accuracy. The *3c* and *p* subscripts refer to the three categories classification and to the pipeline classification, respectively.

Accuracy	PCA + SVM_3c_	PCA + SVM_p_	PCA + LDA_3c_	PCA + LDA_p_	LDA_3c_	LDA_p_	ANN_3c_	ANN_p_	CNN_3c_	CNN_p_	CLARA
$Acc_{E}^{f}$	100.0%	60.0%	87.1%	67.3%	35.5%	26.7%	100.0%	96.4%	100.0%	100.0%	100.0%
$Acc_{G1}^{f}$	4.2%	2.5%	8.3%	2.8%	29.2%	18.6%	4.2%	16.0%	0.0%	20.8%	83.3%
$Acc_{G2G3}^{f}$	75.8%	82.8%	100.0%	100.0%	100.0%	100.0%	100.0%	100.0%	100.0%	100.0%	100.0%
(2) $Acc^{f}$	69.0%	65.7%	82.6%	78.3%	75.5%	72.5%	84.5%	86.1%	83.8%	87.6%	**97.4**%

**Table 2:** CLARA 1D CNN accuracy compared with most common machine learning methods. The classification task aims at recognizing EG1 tumors from G2G3 ones. The first two rows report the per-class accuracy, with the last row showing the mean accuracy.

Accuracy	PCA + SVM	PCA + LDA	LDA	ANN	CLARA (CNN)
$Acc_{EG1}^{1}$	60.0%	67.3%	63.6%	96.4%	100%
$Acc_{G2G3}^{1}$	82.8%	100%	100%	100%	100%
$Acc^{1}$	74.7%	88.3%	87.0%	98.7%	100%

**Table 3:** CLARA 2D CNN accuracy compared with most common machine learning methods. The classification task aims at recognizing E tumors from G1 ones. The first two rows report the per-class accuracy, with the last row showing the mean accuracy.

Accuracy	PCA + SVM	PCA + LDA	LDA	ANN	CNN	SENet	EfficientNet	CLARA (ResNet18)
$Acc_{E}^{2}$	100.0%	100.0%	41.9%	100.0%	100%	100%	100%	100%
$Acc_{G1}^{2}$	4.2%	4.2%	29.2%	16.7%	20.8%	79.2%	66.7%	83.3%
$Acc^{2}$	58.2%	58.2%	36.4%	63.0%	65.4%	90.9%	85.4%	92.73%

## Data Availability

The request for data sets generated in the present study can be agreed and made directly to the corresponding author.
